# Subarachnoid hemorrhage complicated by cerebral venous sinus thrombosis: a quantitative systematic review of cases

**DOI:** 10.3389/fneur.2026.1718666

**Published:** 2026-02-02

**Authors:** Xinchen Ma, Xuan He, Dujuan Sha

**Affiliations:** 1 Department of General Practice, Affiliated Hospital of Shaoxing University, Shaoxing, Zhejiang, China; 2 Department of General Practice, Nanjing Drum Tower Hospital, Affiliated Hospital of Medical School, Nanjing University, Nanjing, China

**Keywords:** cerebral venous sinus, hemorrhage, review, subarachnoid, thrombosis

## Abstract

Subarachnoid hemorrhage (SAH) is increasingly being recognized as a potential complication of cerebral venous sinus thrombosis (CVST), posing challenges in diagnosis and prognosis. We conducted a systematic search of case reports published from 1996 to 2023 in PubMed and Web of Science using the terms “sinus thrombosis, intracranial” and “subarachnoid hemorrhage” and identified 94 cases from 58 articles. Analysis of these cases suggests potential predictors of CVST complicated by SAH, including epilepsy, pregnancy history, abortion history, migraine history, thrombosis in the superior sagittal sinus, and thrombosis involving both the superior sagittal and transverse sinuses. These findings could stimulate further research on the diagnosis and treatment of CVST complicated by SAH.

## 1 Introduction

Cerebral venous sinus thrombosis (CVST) is a series of cerebrovascular diseases caused by various etiologies and features, including the obstruction of cerebrovenous return and impaired absorption of cerebrospinal fluid. It is a rare and underrecognized kind of stroke that accounts for 0.5–1% of all stroke occurrences, yet the fatality rate can reach 10% (1). As a very serious but common encephalopathy, although the main causes of subarachnoid hemorrhage (SAH) are ruptured aneurysms and arteriovenous malformations, cerebral venous sinus thrombosis could be associated with SAH in a few cases (2).

The frequency of CVST in daily practice is increasing, and some CVST cases have been reported to be complicated by SAH. SAH is becoming widely recognized as a possible complication of CVST. The possible pathophysiological mechanisms are as follows. First, cerebral venous thrombosis induces local inflammation, increases vascular permeability, and allows blood to enter the subarachnoid space. Second, venous parenchymal hemorrhagic infarction is a potential complication in patients with CVST and may rupture into the subarachnoid space in some cases. Finally, dural sinus thrombosis extends to the superficial vein, resulting in local venous hypertension accompanied by dilation of the thin and fragile parietal cortical veins and eventual rupture into the SA space (3).

On the other hand, CVST is characterized by a variety of vague clinical symptoms that coincide with those of other disorders, including SAH. The non-specific symptoms affect the early diagnosis and treatment of CVST.

## 2 Methods

### 2.1 Search strategy

**Figure 1:**
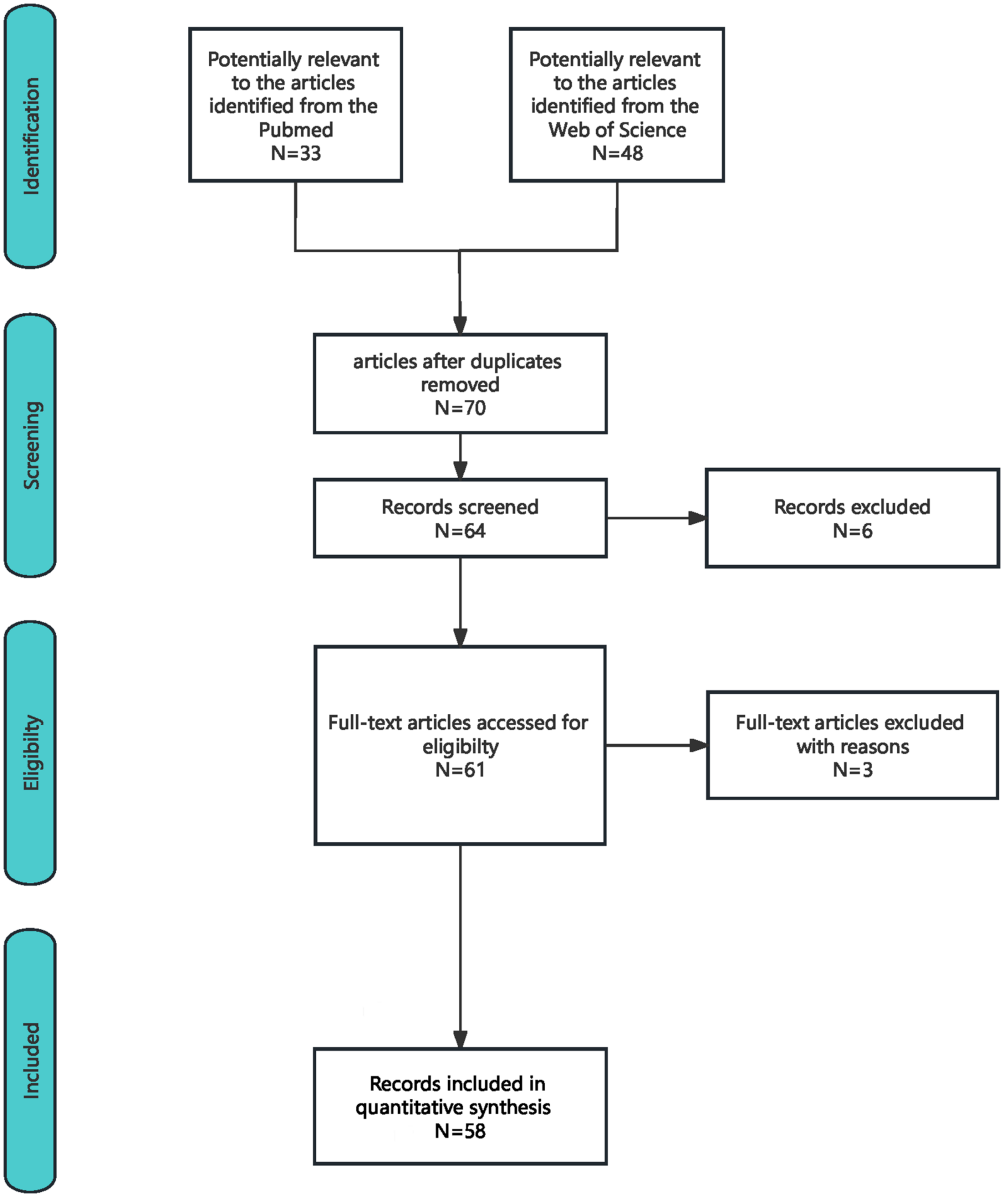
Search strategy flowchart.

We searched for studies collected in PubMed and Web of Science from 1996 to 2023 using the terms “sinus thrombosis,” “intracranial,” and “subarachnoid hemorrhage.” Repeated and inaccessible studies were excluded. We screened case reports and case series reports of CVST complicated by SAH. We excluded emails, studies with only abstracts, reviews, and meta-analyses without case presentations, comments, and letters. The search strategy flowchart is shown in Figure 1.

### 2.2 Data collection

We extracted data on each patient characteristic (age, sex, initial symptoms, possible etiology and risk factors, location of the CVST thrombus, and subsequent treatment) and recorded them in a Microsoft Excel spreadsheet for summary and descriptive analysis. When the information was insufficient or descriptions were unclear, we marked the item as missing in the case information record sheet to be excluded during the statistical process.

## 3 Results

### 3.1 Demography

We extracted data from 94 cases from 58 identified articles. The mean age of the 94 patients was 41.0 ± 12.7 (39.5) years, with the youngest being 14 years and the oldest being 83 years. Of this, 45 were male (45/94, 47.9%, mean age 42.6 years), and 49 were female (49/94, 52.1%, mean age 39.2 years). Among the females, 38 patients were under 50 years old (40.4% of all the reviewed cases) of childbearing age.

### 3.2 Etiology

Of the identified cases, 20 were excluded due to missing risk factors and vague descriptions, leaving 74 cases for etiology statistics. There were 22 young females (22/74, 29.7%) with oral contraceptive and other hormone drug usage etiology (only 4 patients had hormone therapy, but not with enterogastrone), and 12 patients (12/74, 16.2%) had a history of recent pregnancy and abortion. There were 9 patients with a specific embolization status (9/74, 12.2%, including 1 with factor III deficiency, 2 with anticardiolipin antibody syndrome, 1 with activated protein C resistance, 3 with a Factor V Leyden mutation, and 2 with a factor II G20210A mutation). Eight patients (8/74, 10.8%) had a history of migraine, and 7 patients (7/74, 9.46%) had received the influenza vaccine within 1 month before the onset of illness. Increased homocysteine was found in five patients, and five patients suffered from spinal craniocerebral traumatic operations, such as lumbar puncture and craniocerebral trauma. Five patients had a history of thrombotic events, and four patients had a history of autoimmune diseases other than APS (including three cases of Graves’ disease and one case of suspected Behcet’s disease). Four patients had a history of polycythemia. Four patients had gastroenteritis and dehydration. We also counted the single and combined situations of these factors, which are all presented in Supplementary Table S1.

### 3.3 Symptoms and signs

**Figure 2:**
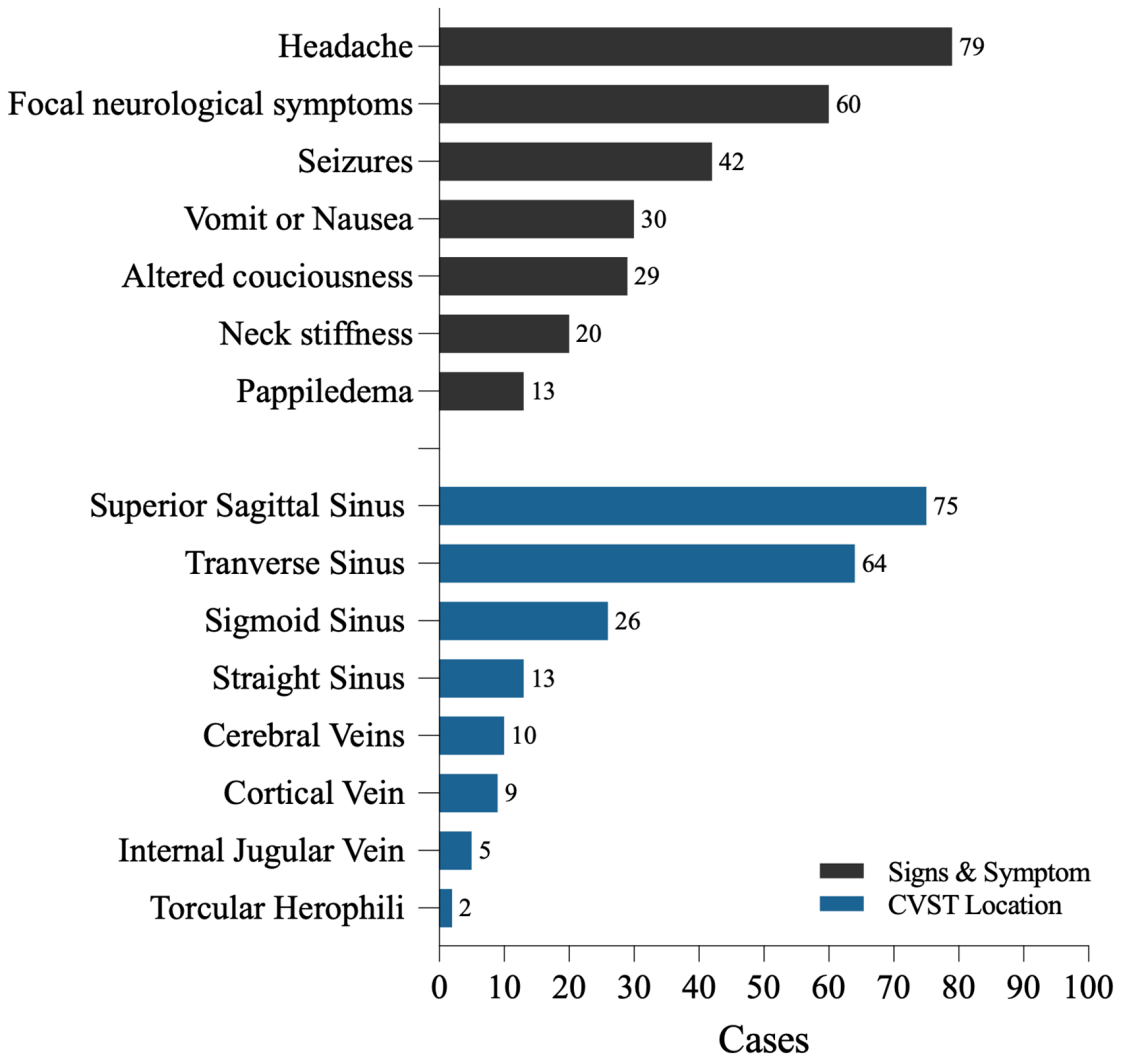
The symptoms and CVST locations of 94 CVST cases with SAH.

Among the 94 patients, 79 reported symptoms of headache (79/94, 84.0%). Among these, 13 were described as thunderclap or lightning-like headaches, 25 had subacute headaches, and 3 had progressive headaches lasting from 3 days to 1 month. Nausea or vomiting occurred in 30 patients (31.6%), and papilledema was observed in 13 patients during physical examination. A total of 84 patients (89.7%) exhibited signs of intracranial hypertension, as mentioned above. Seizures were the initial symptoms in 42 patients, accounting for 44.7% (among patients describing specific types of seizures, 13 had generalized seizures and 11 had partial seizures). A total of 60 patients (60/94, 63.83%) presented with focal neurological symptoms, including hemiparesis, sensory abnormalities, and non-specific language impairments, which may be closely related to dysfunction of the affected venous system in the brain area. A total of 29 patients (29/94, 30.9%) experienced changes in consciousness and mental status, among whom 12 patients were clearly described as having severe impairment of consciousness (delirium, coma, Glasgow Coma Scale (GCS) ≤ 9). A total of 11 patients had mild symptoms, such as drowsiness, irritability, and mild confusion, with GCS scores greater than 9. Neck stiffness or rigidity was observed in 20 (21.3%) patients (Figure 2).

### 3.4 CVST location

There were 76 cases (76/94, 80.9%) of thrombosis in the superior sagittal sinus (SSS), 64 cases (64/94, 68.1%) in the transverse sinus (T), 26 cases (26/94, 27.67%) in the sigmoid sinus (SS), and 13 cases (13/94, 13.8%) in the straight sinus (St). We found a few cases of thrombosis located in the inferior sagittal sinus (ISS) and torcular herophili. SSS and T were simultaneously involved in 45 cases (45/94, 45.8%), and there were 57 cases of multiple thromboses involving two or more sites (57/94, 53.2%) (Table 1; Figure 2).

**Table 1:** CVST locations in 94 CVST cases with SAH.

Age	Sex	CVST Location	References
37	F	SSS + T + SS	de Bruijn et al. (19)
32	F	T + SS	de Bruijn et al. (19)
54	F	SSS	Ohta et al. (20)
36	F	T + SS + CV(c)	Ciccone et al. (21)
60	M	SSS + T + SS	Ra et al. (22)
58	F	T + SS	Sztajzel et al. (23)
38	M	SSS + T + St + CV + IJV + TH	Selim et al. (24)
36	M	SSS + T + St + IJV + CV	Selim et al. (24)
33	F	SSS + CV(c)	Widjaja et al. (25)
22	F	SSS	Widjaja et al. (25)
43	F	SSS + T + St + CV(c)	Widjaja et al. (25)
27	F	SSS + T + IJV	Tidahy et al. (26)
14	M	SSS + St + CV	Adaletli et al. (27)
69	M	SSS + T	Oppenheim et al. (28)
55	F	SSS + T	Oppenheim et al. (28)
32	F	SSS + T	Oppenheim et al. (28)
51	F	SSS + CV(c)	Oppenheim et al. (28)
41	F	SSS	Spitzer et al. (10)
42	M	SSS + T	Spitzer et al. (10)
45	F	SSS + T + SS + St	Zare and Mirabdolbaghi (29)
39	M	SSS + T	Kasuga et al. (30)
44	M	SSS + T	Lin et al. (31)
56	F	SSS + T	Rice et al. (32)
31	F	SSS	Senel et al. (33)
40	M	SSS + T + SS + ISS	Shukla et al. (34)
40	M	SSS + T	Mathew et al. (35)
33	F	T + CV + TH	Wang et al. (36)
53	F	SSS	Jaiser et al. (37)
34	M	SSS	Lai et al. (38)
43	F	SSS + T	Tang et al. (39)
37	F	SSS + T	Tang et al. (39)
46	F	SSS + T	Tang et al. (39)
48	M	SSS + T	Tang et al. (39)
30	M	SSS + T	Tang et al. (39)
23	M	SSS + T	Tang et al. (39)
56	M	SSS + T	Benabu et al. (3)
31	F	SSS	Bittencourt et al. (40)
72	F	T + SS + St + CV	Lee et al. (41)
39	F	St + ISS + CV	Lee et al. (41)
38	F	SSS + T	Hegazi et al. (42)
52	F	SSS + T + SS + St	Kato et al. (43)
40	M	SSS + T + SS	Sharma et al. (44)
32	M	SSS + T	Panda et al. (11)
50	M	SSS + T + St	Panda et al. (11)
27	F	SSS + T + St	Panda et al. (11)
33	M	SSS	Panda et al. (11)
32	F	SSS	Panda et al. (11)
25	M	SSS	Panda et al. (11)
38	M	SSS + T	Panda et al. (11)
39	M	SSS	Panda et al. (11)
38	M	SSS + T + SS + St + ISS	Panda et al. (11)
59	M	SSS + T	Sharma et al. (45)
70	M	SSS	Field et al. (46)
83	M	T	Oda et al. (47)
22	M	SSS + T	Oz et al. (48)
42	F	T + SS	Saya et al. (49)
36	M	SSS + T + SS	Saya et al. (49)
48	M	SSS	Sahin et al. (13)
24	F	SSS + CV(c)	Froehler (50)
30	M	T + SS + CV	Kulkarni et al. (51)
22	F	SSS + T	Mathon et al. (52)
42	M	SSS + T + SS	Anderson et al. (53)
46	M	SSS + T + SS	Hassan et al. (54)
35	M	SSS	Hassan et al. (54)
40	F	SSS + CV(c)	Bansal et al. (55)
58	M	SSS	Kathib et al. (56)
45	M	T	Fu et al. (57)
45	M	T + SS	Liang et al. (58)
38	M	SSS	Uniyal et al. (59)
58	M	SSS + St	Abbas et al. (60)
44	F	T + SS + IJV	Amer et al. (61)
20	F	SSS	Han et al. (62)
57	F	SSS	Sun et al. (63)
32	M	SSS + T + CV(c)	Mehta et al. (64)
25	M	SSS + CV(c)	Mehta et al. (64)
62	M	SSS + CV(c)	Bérezné et al. (65)
54	F	SSS + CV	D’Agostino et al. (66)
58	F	T	Gajurel et al. (67)
25	F	T	Kumar et al. (68)
45	M	SSS	Syed et al. (69)
22	F	SSS + T + SS	Wolf et al. (12)
46	F	SSS + T + SS	Wolf et al. (12)
36	F	SSS + T	Medeiros et al. (70)
28	F	SSS + T + SS	Medeiros et al. (70)
49	F	SSS + T + SS	Medeiros et al. (70)
30	F	SSS + CV	Medeiros et al. (70)
28	F	SS + IJV	Medeiros et al. (70)
44	M	T	Medeiros et al. (70)
40	F	SSS + T + SS	Medeiros et al. (70)
43	F	SSS	Medeiros et al. (70)
38	F	T	Medeiros et al. (70)
32	F	SSS + T + SS + St + IJV	Medeiros et al. (70)
47	M	SSS + T	Medeiros et al. (70)
39	M	SS + T + CV	Sakashita et al. (71)

SSS, superior sagittal sinus; T, transverse sinus; St, straight sinus; SS, sigmoid sinus; ISS, Inferior sagital sinus; IJV, Internal jugular vein; CV, Cerebral veins; CV, Cerebral veins including Galen, Labbe, and Trolard; CV(c), Cortical Vein; TH, torcular herophili.

### 3.5 Treatment

Seventy-four patients underwent anticoagulant therapy. During follow-up, 56 patients achieved complete recovery, 8 showed partial recovery, and 6 were discharged with partial remission but were lost to follow-up. One patient exhibited no improvement, while three experienced rapid deterioration and died. Additionally, one patient had a short-term recurrence of cerebral venous thrombosis. Moreover, among the patients who received subcutaneous low-molecular-weight heparin followed by oral anticoagulation, all achieved complete remission, except for one who was lost to follow-up (Supplementary Table S2).

## 4 Discussion

There are a few reports of concurrent subarachnoid hemorrhage in CVST. A study of baseline characteristics of patients with CVST in the National Readmissions Database (NRD) found that the average age of patients was 46.8 years, with a predominance of females (65%) (4). Our retrospective analysis yielded similar results regarding the average age of the patients compared with the above research. However, among the patients with CVST and concurrent SAH included in our review, the sex distribution was close to 1:1. Reproductive-age females did not constitute the majority of patients in our review of concurrent SAH cases, although they are considered the main population at risk for CVST (5). Lin et al. (6) found that compared to patients of other ages and genders, reproductive-age females with CVST had better outcomes and were less likely to experience severe complications, including intracranial hemorrhage; the mechanism behind this phenomenon remains unclear.

Our review of CVST combined with SAH found a relatively higher proportion of patients with recent pregnancy history than the susceptibility factors described by Ferro et al. (33.3% vs. 21%) (7). This may be attributed to hormonal changes during pregnancy, especially the elevation of estrogen levels, which can affect the stability of blood vessel walls, making them more fragile and prone to damage. Increased blood volume and cardiac output during pregnancy may subject the vascular system to additional stress. These changes may exacerbate the pathological condition of cerebral veins already affected by thrombosis. Other etiologies or risk factors did not show any additional noteworthy characteristics.

Thunderclap headache, often known as lightning strike headache, is the primary symptom of subarachnoid hemorrhage (SAH) (8). However, in the cases of cerebral venous sinus thrombosis (CVST) accompanied by SAH that we examined, less than 20% of the patients had this distinctive headache. This result implies that CVST-induced SAH rarely results in thunderclap headaches. This could be due to the different causes of SAH; ruptured aneurysms account for approximately 90% of SAH cases (8), and sudden and intense bleeding from aneurysmal rupture frequently displays as thunderclap headaches. CVST, a less prevalent cause of SAH, is characterized by a progressive pathophysiological mechanism that results in bleeding. In cases where CVST causes SAH, rupture of the venous sinuses and superficial veins directly into the subarachnoid space often relieves venous congestion and surrounding edema, resulting in diffuse bleeding in the subarachnoid space. Consequently, local intracranial pressure may be alleviated, leading to milder headaches than those caused by ruptured aneurysms. Among all patients, 16 presented with isolated headaches as the initial symptom, without focal neurological symptoms, seizures, or impaired mental status. The initial headaches in these patients are often confused with migraines, posing a challenge for clinical diagnosis and making them prone to misdiagnosis.

Lindgren et al. (9) conducted a large-scale study on the association between CVST and seizures, finding that 34% of 1,281 patients experienced seizures within 1 week after cerebral venous thrombosis formation. In contrast, we noticed that 44.7% of patients with CVST combined with SAH experienced seizures at onset, which represents an increased proportion compared to the aforementioned studies. Based on this comparison, we propose that seizures may be related to CVST complicated by SAH, especially when SAH occurs in the cerebral convexity region, which may directly stimulate the cerebral cortex and trigger seizures. Spontaneous cerebral convex subarachnoid hemorrhage (cSAH) is a rare and distinct subtype of subarachnoid hemorrhage and is an important subtype of non-aneurysmal subarachnoid hemorrhage (2). The characteristic feature of this condition is the presence of bleeding limited to one or more sulci of the cerebral convexity, without involving the adjacent brain parenchyma, longitudinal fissures, basal cisterns, or ventricles. When SAH is caused by cerebral venous sinus thrombosis, cSAH is the most common presentation (10). In a study by Panda et al. (11), evidence of concurrent SAH was found in 10 of 233 patients with CVST, all of whom had cSAH. This suggests that the presence of cSAH with bleeding not involving the basal cisterns implies that bleeding is induced by CVST. Our analysis also revealed that 88% of patients with seizures had concurrent cSAH, possibly due to the direct stimulation of the cerebral cortex by cSAH bleeding. Conversely, among the eight patients with SAH limited to the perimesencephalic cisterns and ventricles, only one patient experienced seizures, confirming the association between seizures in patients with CVST and cSAH.

The precise etiology of subarachnoid hemorrhage (SAH) in cerebral venous thrombosis (CVT) remains unclear. The most plausible explanation lies in the anatomical characteristics of the bridging superficial veins. These veins traverse the subarachnoid space before draining into the dural sinuses and are characterized by thin walls, absence of muscular fibers, and lack of valves. This unique structure grants the cerebral venous system significant capacitance while rendering it susceptible to blood flow reversal during thrombosis (12). Under conditions of venous hypertension, such fragile vessels are prone to rupture, leading to blood extravasation into the subarachnoid space (12). Furthermore, cortical vein thrombosis may occur as a result of retrograde propagation of dural sinus thrombosis (13). Another potential mechanism may involve an inflammatory response induced by CVST, which could increase vascular permeability and allow blood to leak into the subarachnoid space. Additionally, hemorrhagic venous infarction might lead to secondary rupture into the subarachnoid spaces, although this scenario remains infrequently documented in the literature (13).

Multiple studies have shown that the superior sagittal sinus (SSS) is the most common site of CVST, followed by the transverse sinus (TS) and sigmoid sinus (5, 7, 14). SSS accounts for 62–80% of cases, while TS is involved in 38–86% of cases (14). Thrombus formation affects multiple venous sinuses in approximately 75% of cases, with the most common combination being SSS + TS, affecting approximately 30% of patients with CVST simultaneously (14). In reviewing the thrombus sites in CVST complicated by SAH, SSS remains the most prevalent, with the proportion of SSS thrombosis being higher than that reported by Ferro et al. in their study of the CVST population (80.9% vs. 62%). The second most common site is the transverse sinus. However, the sigmoid sinus takes third place, accounting for 27.6%, whereas Ferro et al. reported that the incidence of sigmoid sinus involvement in CVST is less than 10% (7). The proportion of cases involving two or more venous sinuses is lower than that reported by Ferro et al. in their study of the CVST population (53.2% vs. 75%), while involvement of both SSS and TS is 45.8%, which is also the most common combination (5). Therefore, if imaging shows thrombosis in the SSS and both the SSS and TS, vigilance should be given regarding the risk of concomitant SAH.

Systemic anticoagulation serves as the first-line treatment for CVST due to its well-established efficacy and safety profile. Notably, the presence of hemorrhage in CVST does not contraindicate anticoagulant therapy; once imaging confirms CVST, anticoagulation should be initiated regardless of whether intracerebral bleeding is present (15, 16). Regarding anticoagulant selection, standard management of cerebral venous thrombosis (CVT) typically begins with parenteral anticoagulation using unfractionated or low-molecular-weight heparin, followed by long-term oral therapy with vitamin K antagonists (VKAs) (15, 16). In practice, for patients requiring continued anticoagulation after discharge—particularly when regular coagulation monitoring is challenging—*dabigatran* may be considered as a maintenance option. Among the cases reviewed, eight patients received *dabigatran* orally for several months post-discharge, all of whom exhibited favorable outcomes. Direct oral anticoagulants (DOACs)， including factor Xa inhibitors (e.g., *rivaroxaban, edoxaban*) and the factor IIa inhibitor *dabigatran*, are increasingly used off-label for CVT, with studies suggesting comparable efficacy and safety to warfarin (17) and potentially superior recanalization rates versus vitamin K antagonists (18). However, evidence remains limited due to disease rarity, underscoring the need for international multicenter studies to establish DOACs’ role in CVST management.

## 5 Conclusion

Based on the results of the above case analysis, seizures, obstetric history, migraine history, thrombosis in the superior sagittal sinus, and thrombosis involving both the superior sagittal and transverse sinuses may be potential risk factors for SAH in patients with CVST. Patients with CVST with these characteristics should be vigilant about the risk of developing SAH, and targeted interventions should be implemented to improve patient outcomes. More diverse-sample case–control and cohort studies are needed. This will enable timely diagnosis and intervention to prevent the severe consequences of SAH in patients with CVST with the aforementioned conditions, as well as advise anticoagulant choice and timing.

## 6 Limitations

As it is a rare disease and its complications, data from fewer than 100 cases were collected. Our study used secondary information, so we could not directly contact the patients, and some missing information in the cases affected the accuracy of the analysis results. Consequently, the information could only be analyzed descriptively.

## Data Availability

The original contributions presented in the study are included in the article/Supplementary material, further inquiries can be directed to the corresponding author.
