# Supplementary material for: Subarachnoid hemorrhage complicated by cerebral venous sinus thrombosis: a quantitative systematic review of cases
Source: Front Neurol. 2026 Feb 2;17:1718666. doi: 10.3389/fneur.2026.1718666 (PMC12907161; doi:10.3389/fneur.2026.1718666)
Supplement: Supplementary file 1 [file Data_Sheet_1.pdf]

Table S1. Risk factors for CVST with SAH

Risk factors	Case（n）	Reference
Single risk factors	40	
① OC and other hormone drug	10	[5],[10][1],[10][3],[19],[30][3],[36],[43],[59][1],[59][7],[59][10]
② Recent pregnancy and abortion	3	[30][5],[50], [56]
③ Specific embolization status	2	[29][6], [59][6]
④ History of migraine	2	[13][1],[44]
⑤ Recent influenza vaccination	5	[52][1],[54],[57],[58][1],[58][2]
⑥ Meningitis	4	[12],[17],[18],[32]
⑦ Craniocerebral traumatic operations	2	[35], [59][11]
⑧ History of autoimmune disease except APS	4	[7],[16],[27],[46]
⑨ Raised homocysteine	2	[9][2],[3]
⑩ Gastroenteritis and dehydration	1	[48]
⑪ Polycythemia	2	[59][2], [59][4]
⑫ History of thrombotic event	1	[41]
⑬ Family history of deep vein thrombosis	1	[51]
⑭ Acute lymphoid leukemia	1	[59][8]
In good health before	9	[21],[22],[23],[24],[28],[31], [42][2],[47],[55]
Negative underlying etiology	6	[27][1],[29],[32],[37],[42][1],[49]
Multiple risk factors	19	
①, ②	2	[59][2], [59][4]
①, ④	1	[4][2]
①, ⑦	1	[26]
②, ④	1	[15]
②, ⑫	1	[59][3]
④, ③	1	[38]
⑨, ⑪	1	[30][8]
⑪, Inflammation outside CNS	1	[9][1]
⑤, ⑩	1	[53]
①, ②, ⑩	1	[6]
①, ②, ③	1	[59][5]
①, ②, ⑫	1	[59][9]
①, ②, ⑬	1	[13][4]
①, ③, ⑫	1	[8]
①, ③, ⑨	1	[11]
①, ④, ⑫	1	[13][2]
①, ⑨, Inflammation outside CNS	1	[13][3]
⑤, ④, ③	1	[52][2]

OC, oral contraceptives, and the following OC in multiple risk factors omits other hormone drug; APS, anticardiolipin antibody syndrome; CNS, central nervous system.
