# Supplementary material for: Subarachnoid hemorrhage complicated by cerebral venous sinus thrombosis: a quantitative systematic review of cases
Source: Front Neurol. 2026 Feb 2;17:1718666. doi: 10.3389/fneur.2026.1718666 (PMC12907161; doi:10.3389/fneur.2026.1718666)
Supplement: Supplementary file 2 [file Data_Sheet_2.pdf]

Table S2. Treatment and Outcome of CVST cases with SAH

Age	Sex	Treatment	Outcome	Reference	Age	Sex	Treatment	Outcome	Reference
37	F	-	-	De Bruijn[4]	25	M	AC	CR	Panda[30]
32	F	-	-	De Bruijn[4]	38	M	AC	CR	Panda[30]
54	F	Oral	CR	Ohta[5]	39	M	AC	CR	Panda[30]
36	F	-	CR	Ciccone[6]	38	M	AC	CR	Panda[30]
60	M	Intravascular thrombolysis	CR	Ra CS [7]	59	M	LMWH→Oral	PR (No follow-up)	Sharma S[32]
58	F	LMWH→Oral	CR	Sztajzel[8]	70	M	IV UFH→Warfarin	CR	Field DK[33]
38	M	IV UFH+ jugular vein stent	CR	Selim M[9]	83	M	-	-	Oda S[34]
36	M	t-PA + LMWH	PR	Selim M[9]	22	M	Only antiedema	-	Oz O[35]
33	F	AC	CR	Widjaja[10]	42	F	AC	PR (No follow-up)	Saya M[36]
22	F	Intravascular thrombolysis	CR	Widjaja[10]	36	M	AC	PR (No follow-up)	Saya M[36]
43	F	IV UFH→Warfarin	CR	Widjaja[10]	48	M	AC	PR (No follow-up)	Neslin Sahin [37]
27	F	IV UFH	CR	Tidahy[11]	24	F	SC UFH + Intravascular thrombolysis + AP	CR	Froehler [38]
14	M	AC	CR	Adaletli[12]	30	M	IV UFH→ VKA + Ventriculoperitoneal shunt	Death	Kulkarni [39]
69	M	IV UFH	CR	Oppenheim[13]	22	F	AC	CR	Mathon [40]
55	F	LMWH	CR	Oppenheim[13]	42	M	AC	CR	Anderson [41]
32	F	IV UFH	CR	Oppenheim[13]	46	M	IV UFH→Oral	CR	Hassan[42]
51	F	AC	CR	Oppenheim[13]	35	M	SC UFH→VKA	PR (No follow-up)	Hassan[42]
41	F	IV UFH→VKA	CR	Spitzer[14]	40	F	Ventriculoperitoneal shunt	Death	Bansal [43]
42	M	IV UFH→VKA	CR	Spitzer[14]	58	M	LMWH→Oral	CR	Kathib [44]
45	F	Warfarin	PR	Zare[15]	45	M	LMWH→Warfarin	CR	Fu [45]
39	M	IV UFH→VKA	CR	Kasuga [16]	45	M	AC	CR	Liang [46]
44	M	IV UFH→VKA	CR	Lin JH [17]	38	M	LMWH→Oral	CR	Uniyal [47]
56	F	AC	CR	Rice H [18]	58	M	LMWH→Rivaroxaban	CR	Abbas [48]
31	F	AC	CR	Senel A [19]	44	F	LMWH→Warfarin	CR	Amer [49]
40	M	-	-	Shukla R [20]	20	F	AC	CR	Han [50]
40	M	LMWH→Oral	CR	Mathew T[21]	57	F	No AC or thrombolysis	CR	Sun [51]
33	F	EBA	Relapse	Wang YF[22]	32	M	-	Death	Mehta [52]
53	F	IV UFH→LMWH→Warfarin	CR	Jaiser SR[23]	25	M	IV UFH	Death	Mehta[52]
34	M	IV UFH→Warfarin	PR	Lai NK [24]	62	M	IV UFH + GC	Death	Bérezné [53]
43	F	-	-	Tang PH [25]	54	F	-	Death	D'Agostino [54]
37	F	-	-	Tang PH [25]	58	F	Dabigatran	CR	Gajurel[55]
46	F	-	-	Tang PH [25]	25	F	-	-	Kumar [56]
48	M	-	-	Tang PH [25]	45	M	IV UFH→VKA	CR	Syed [57]
30	M	-	-	Tang PH [25]	22	F	LMWH→Dabi	CR	Wolf [58]
23	M	-	-	Tang PH [25]	46	F	LMWH→Dabi	CR	Wolf [58]
56	M	AC	CR	Benabu[3]	36	F	Warfarin	CR	Medeiros[59]
31	F	AC	PR	Bittencourt [26]	28	F	Warfarin	CR	Medeiros[59]

72	F	GC +AC	PR	Lee J [27]	49	F	Warfarin	PR	Medeiros[59]
39	F	IV UFH + AP + Nimodipine	PR	Lee J[27]	30	F	Warfarin	PR	Medeiros[59]
38	F	LMWH→Warfarin	CR	Hegazi MO[28]	28	F	Warfarin	CR	Medeiros[59]
52	F	IV UFH→Warfarin	CR	Kato Y [29]	44	M	IV UFH→Warfarin	PR	Medeiros[59]
40	M	AC	PR (No follow-up)	Sharma B[31]	40	F	Warfarin	-	Medeiros[59]
32	M	AC	CR	Panda[30]	43	F	Warfarin	No Recovery	Medeiros[59]
50	M	AC	CR	Panda[30]	38	F	No treatment	Death	Medeiros[59]
27	F	AC	CR	Panda[30]	32	F	Warfarin	-	Medeiros[59]
33	M	AC	CR	Panda[30]	47	M	Warfarin	CR	Medeiros[59]
32	F	AC + Intravascular thrombolysis	CR	Panda[30]	39	M	IV UFH→Warfarin	CR	Sakashita [60]

AC, unknown drug-induced anticoagulants; GC, glucocorticoids; AP, antiplatelet; CR, complete remission; PR, partial remission; LMWH, low molecular weight heparin; IV UFH, intravenous heparin; SC UFH, subcutaneous unfractionated heparin; Oral anticoagulants; VKA, vitamin K antagonists; EBA, extended bifrontal pericardiotomy; – unknown or not mentioned
